# Comparative Demography of the Spider Mite, *Tetranychus ludeni*, on Two Host Plants in West Africa

**DOI:** 10.1673/031.006.4901

**Published:** 2006-12-31

**Authors:** Etienne Adango, Alexis Onzo, Rachid Hanna, Pierre Atachi, Braima James

**Affiliations:** ^1^ Biological Control Centre for Africa, International Institute of Tropical Agriculture, 08 B.P. 0932, Cotonou Bénin, West Africa; ^2^ Institut National des Recherches Agricoles du Bénin (INRAB), Laboratoire de Défense des Cultures (LDC) B.P. 128, Porto-Novo Bénin, West Africa; ^3^ Université d'Abomey-Calavi, Faculté des Sciences Agronomiques (FSA/UAC), 01 B.P. 526 Cotonou Bénin, West Africa

**Keywords:** Acari, Tetranychidae, vegetable crops, *Amaranthus cruentus*, *Solanum macrocarpon*, life table parameters

## Abstract

It is well recognized that the quality of host plants affects the development and survival of plant-feeding arthropods. The effects of two leafy vegetable crops, amaranth, *Amaranthus cruentus* L. (Caryophyllales: Amaranthaceae) and nightshade, *Solanum macrocarpon* L. (Solanales: Solanaceae) were examined on the development and demographic parameters of the spider mite, *Tetranychus ludeni* Zacher (Acari: Tetranychidae). This mite was recently identified as a pest of the two leafy vegetables which are widely used in West Africa. The experiments were conducted at the International Institute of Tropical Agriculture, Benin, West Africa, in a growth chamber at 27°C, 70% ±10% RH and 12:12 (L:D). Immature development of *T. ludeni* was shorter on *A. cruentus* than on *S. macrocarpon*, whereas female longevity was the same on the two vegetable crops. Total fecundity per female was higher on *A. cruentus* than on *S. macrocarpon*, largely due to longer survival of adult female *T. ludeni* on the former; however, no differences were observed in the daily fecundity of *T. ludeni* on the two plant species. The comparison of intrinsic rates of natural increase (*r_m_*), the net reproductive rates (*R_o_*) and the survival rates of adult stage of *T. ludeni* on the two vegetable crops suggests that *T. ludeni* performs better on *S. macrocarpon* than on *A. cruentus*. Reasons for the lower rate of population growth observed on amaranth should be studied in more details as this could be used in IPM strategies such as intercropping to reduce pest density and in developing biopesticides for use against *T. ludeni* in vegetable farms in Africa.

## Introduction

It is now well recognized that host plant quality can affect several life-history characteristics of their herbivores, by impairing growth, lowering resistance to disease and reducing fecundity (Price et al. 1980). Alteration of population growth results from changes in fecundity, survivorship and development rates of the herbivore (Walde 1995). Chemical traits such as toxins, digestibility-reducers and nutrient balance, or physical traits such as pubescence and tissue toughness, vary from one host plant to another, with different effects on population levels of the herbivores.

In the prevailing vegetable cropping systems in sub-Saharan Africa, several vegetable crop species are grown in close proximity in small lots. The crops are attacked by several pest species (Meyer 1996), and the proximity of plots probably favors dispersal of pests from one crop species to the other. In such polyculture, attacks by herbivorous arthropods can be influenced by host plant quality. Generally, the herbivore encounters plants of variable quality and departs from the poorer-quality plants more rapidly (Andow 1991). Such behavior can be very important for IPM strategies through creation of spatial/cropping designs (e.g. intercropping) that reduce herbivore attacks, or through resistance that develops as a synergic effect, for example when a plant is included that is acceptable to the mite, but its development is poor, resulting in a decrease in pest density (Greco et al. 2006). This, therefore, motivates our interest in determining whether or not the pest species perform differently on those vegetable crops.

Mites (Order Acarina) in the family Tetranychidae are among the most important crop pests worldwide. The subfamily Tetranychinae includes a number of economically significant species of which *Tetranychus urticae* Koch and *T. ludeni* Zacher are the most important on many vegetable crops (McKinlay et al. 1992; Bordat and Goudegnon 1991; Meyer 1996; Adango 2005). These two mite pests are considered significant constraints to vegetable production in Benin, West Africa (Bordat and Goudegnon 1991; Adango 2005). *Tetranychus ludeni* is widespread in the tropics and has been recorded from over than 300 plant species worldwide (Bolland et al. 1998 cited by Zhang 2002). In India, *T. ludeni* commonly occurs on many cultivated crops, especially on vegetable crops, causing substantial losses, with the highest damage on eggplant and okra (Reddy 2001). Control of these spider mites have been greatly aggravated by their marked ability to develop resistance to a range of chemicals (Cranham and Helle 1985; Van de Vrie 1985; McKinlay et al. 1992), which has created critical situations for practical pest control. In Australia, Heron et al. (1998), observed that both *T. urticae* and *T. ludeni* on cotton were resistant to all organophosphates tested. In Benin, organophosphates and pyrethroids are the most commonly used pesticides on vegetable farms (Adango 2005) with the potential for induction of resistance in mites.

To overcome the problems of resistance development in spider mites, the search for more durable crop protection solutions based on IPM systems in vegetable farms is necessary (Cranham and Helle 1985). However, one of the key conditions for establishing an efficient and sustainable IPM strategy is the detailed knowledge of the pest through its biology, ecological requirements and associated natural enemies (Ochoa et al. 1994; Reckhaus 1997).

Reproduction in spider mites is very sensitive to a wide variety of intrinsic and extrinsic conditions. Intrinsic factors that influence life table parameters of tetranychid mites include mite strain and level of inbreeding, colony density, age of females and of the population, fertility status of the females and various behavioural parameters (Wrensch 1985). Extrinsic factors include temperature, humidity, light, predation level, intra- and interspecific competition, and also various host plants features, such as strain, plant and soil nutrition and plant age.

Amaranth, *Amaranthus emeritus* L. (Caryophyllales: Amaranthaceae) and nightshade, *Solanum macrocarpon* L. (Solanales: Solanaceae), locally known as ‘Gboma’, are respectively the first and second most commonly grown and consumed leafy vegetable crops species in southern Benin (Hounkpodoté and Tossou 2001) because of their high nutritional value. Amaranth is very rich in micronutrients such as carotene, vitamin C, iron, calcium, and lysine, an essential amino-acid that is lacking in cereals whereas nightshade is very rich in sugar, protein and vitamin C (Adango, 2005). Field observations in Benin showed that they are both susceptible to attacks by tetranychid mites mainly *T. urticae* and *T. ludeni* (Adango 2005). Whereas a large body of work is available on the biology of *T. urticae*, much less is known about *T. ludeni*, particularly in Africa.

In the present study we examine, in a series of laboratory experiments, how life-history characteristics of this mite pest vary between the two crop species. This article is the first of a series of studies on the biology and management of mites attacking leafy vegetables in West Africa, an area that has not been addressed at all.

## Materials and methods

### The spider mites

Colonies of *T. ludeni* were initiated with individuals collected on the Ethiopian nightshade, *Solanum aethiopicum* L. (Solanales: Solanaceae), in a field at the International Institute of Tropical Agriculture (IITA), Cotonou Station in Benin. These mite colonies were maintained indoors on potted plants of *S. aethiopicum* at 26 ± 1°C and 65–85% RH for 2 weeks (Gnanvossou et al. 2003) at IITA before the start of the experiments. Plots of *S. macrocarpon* and *A. cruentus* were also installed at the IITA-Cotonou station, separately in an open field that had been fallow for more than four years. One week after planting, chicken droppings were incorporated into each plot to increase its fertility. Two to four weeks after planting, clean leaves were collected that were used to produce the leaf discs used in the experiments.

### Development of immature stages

To determine the developmental time of immature stages of *T. ludeni* on each of the two vegetable crops, a cohort of 80 eggs deposited during 24 hours in the rearing unit on leaf discs (50 mm in diameter) cut from *S. aethiopicum* were transferred singly using a camel hair brush onto fresh leaf discs (22 mm in diameter), cut from each of *A. cruentus* and *S. macrocarpon*, respectively. These leaf discs were kept on water saturated cotton wool in Petri dishes (15 cm in diameter). Water was added each day as needed to ensure adequate moisture for the leaf discs and to maintain a water barrier to restrict mite movement. These rearing units were kept in a growth chamber at 27°C, 70% ± 10% RH and 12:12 (L:D). Leaf discs were replaced twice weekly to maintain freshness of leaves, thereby preserving host-plant attributes. Observations were made everyday at 06:00 hours and 18:00 hours and the developmental stage reached by each individual was recorded until all adults emerged. Individuals that escaped from leaf discs were not included in the analysis.

**Table 1.**
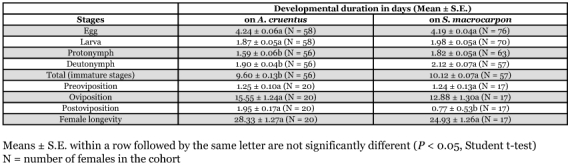
Effects of host plant on the duration of immature and adult development of *T. ludeni* females at 27° C and 70% RH.

### Population growth

To determine the life table characteristics of the herbivorous mite on each of the two vegetable crops, 25 deutonymphal (2 nymphal stage) females (see Bonato et al. 2000; Bounfour and Tanigoshi 2001; Gotoh and Gomi 2003) were randomly selected from the populations initiated for the development study. To ensure mating, two adult males, obtained from the mother colony, were placed with each newly emerged adult female on the leaf disc. The number of eggs deposited by each female was recorded daily until all the females died. Ovipositing females were transferred to new leaf discs every day. The eggs obtained from each female were cultured to determine their hatchability, the survival rates of the immature stages and the sex ratio of the adults expressed as the percentage of females in the progeny.

### Statistical analysis

The Student t-test in SAS (SAS 2003) was used to compare developmental times and oviposition rates between the two crop species. Egg viability was also compared between the two host-plants using the Student t-test on transformed (arcsine square root) proportions of hatched eggs. Homogeneity of variances was tested with the Bartlett's test in SAS. Life table parameters were estimated with the method described by Andrewartha and Birch (1954) and calculated using Jacknife computer program developed by Maia et al. (2000).

**Table 2.**
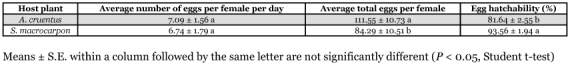
Comparison of egg production and egg hatchability on *A. cruentus* and *S. macrocarpon*

## Results

### Developmental time of immature stages

Except for egg and larval stages that had the same duration on the two vegetable crops, the other life stages were shorter on *A. cruentus* than on *S. macrocarpon* (Table 1; df = 111, *t* = 0.69; *P* = 0.4936 for egg; df = 111, *t* = -1.67, *P* = 0.0977 for larva; df = 111, *t* = -3.21, *P* = 0.0017 for protonymph; df = 111, *t* = -3.21, *P* = 0.0017 for the deutonymph). Similarly, total duration of development from egg to adult was also shorter on *A. cruentus* than on *S. macrocarpon* (Table 1; df = 111, *t* = -3.58, *P* = 0.0005). On both vegetable crops, the longest stage was the egg that had a developmental time that was generally more than two fold longer than any of the other stages.

### Population growth statistics

**Figure 1.**
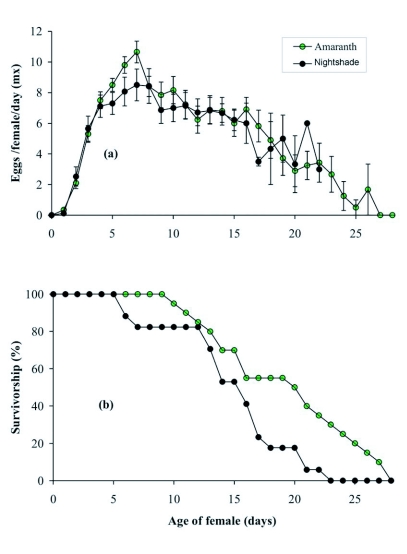
Fecundity **(a)** and age-specific survival **(b)** of *T. ludeni* on two host plants. Day O is the day on which the females became adults. Bars on the fecundity curves represent standard errors of the means.

The longevity of adult female *T. ludeni*, and the length of the pre-oviposition and oviposition periods did not differ significantly between the two vegetable crops tested; however, *T ludeni* had a longer post-oviposition period on *A. cruentus* than on *S. macrocarpon* (Table 1; *P* < 0.05). The total number of eggs laid per female was lower on *S. macrocarpon* than on *A. cruentus* (Table 2; *P* < 0.05); however, daily egg production was similar on the two vegetable crops (Table 2). Egg production rapidly reached its peak, followed by a steady decline seven days after the first egg was laid on the two vegetable crops (Figure 1a). Peak fecundities recorded were 10.7 and 9.9 eggs on *A. cruentus* and *S. macrocarpon*, respectively. Egg viability was higher on *S. macrocarpon* than on *A. cruentus*, with 93.6% and 81.6% hatching on *S. macrocarpon* and *A. cruentus*, respectively (Table 2; *P* < 0.05).

**Table 3.**
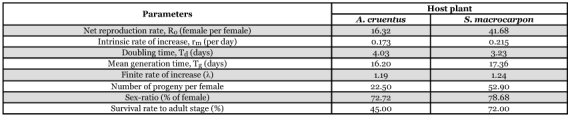
Effect of host plant on the life table parameters of *T. ludeni*.

Survival of adult female *T. ludeni* decreased considerably on *A. cruentus* after nine days whereas on *S. macrocarpon*, the decrease began after five days and became more pronounced after 12 days (Fig. 1b). The survival rate reached 50% on *S. macrocarpon* after 14 days, and on *A. cruentus* after 16 days (Figure 1b).

Life table parameters are presented in Table 3. These results showed that parameters such as the intrinsic rate of natural increase (*r_m_*), net reproductive rate (*R_o_*), female progeny, and the survival rate at adult stage of *T. ludeni* differed between the two host plants. Parameters such as *r_m_*, *R_o_* and female progeny were respectively 1.24, 2.55 and 2.35 fold higher on *S. macrocarpon* than on *A. cruentus*. Similarly, the survival rate of immatures to the adult stage was 72% on *S. macrocarpon* while it was 45% on *A. cruentus*; and doubling time was shorter on *S. macrocarpon* than on *A. cruentus*.

**Figure 2.**
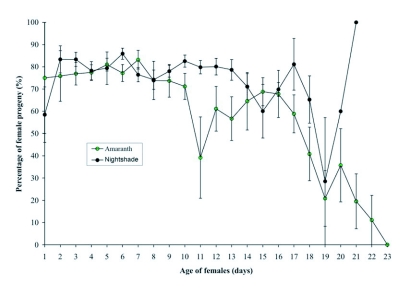
Female production as a function of age of reproductive *T. ludeni* females feeding on the two host plants. Bars represent standard errors of the means.

The temporal fluctuations of the percentage of female progeny (Figure 2) against the age of parents showed that the proportion of females in the progeny was overall higher on *S. macrocarpon*, than on *A. cruentus*. The production of females seems constant on *S. macrocarpon* with a trough only on day 19, followed immediately by a rapid increase at the end of the experiment. On *A. cruentus*, the production of females showed an overall decreasing trend with a trough around day 11 after which it reached a peak before decreasing, starting from day 16 to the end of the experiment.

## Discussion

Our study shows that *T. ludeni* feeds, survives and develops on the two vegetables *A. cruentus* and *S. macrocarpon*, and that host type can greatly affect its development, fecundity and life-table parameters. Indeed, the results showed that the host plant had substantial effects on the intrinsic rate of natural increase (*r_m_*), the net reproductive rate (*R_o_*), female progeny, and the survival of the adult stage.

Developmental times reported in this study on the two vegetables are similar to those reported by other authors. With the same spider mite species, mean duration of developmental stages recorded on brinjal and South African cucurbit were 9.24 and 9.91 days, respectively (Puttaswamy 1980). On *Phaseolus vulgaris* L., mean duration of developmental stages for female *T. ludeni* was 9.98 days (Moros and Aponte 1994), whereas on cotton it was 9.79 days for females and 9.50 for males at 28°C (da Silva 2002). Moros and Aponte (1994), and da Silva (2002) also found that the duration of the egg stage is the longest of all other life stages of *T. ludeni*.

Adult longevity and daily oviposition rate of *T. ludeni* were similar on the two vegetable crops tested, and close to those recorded for the same mite species on *P. vulgaris* (Moros and Aponte 1994). Total fecundity, however, was higher on *A. cruentus* than on *S. macrocarpon*. The larger quantity of eggs and the shorter juvenile developmental times noted on *A. cruentus* may be due to its higher nitrogen content in comparison with *S. macrocarpon* (Schippers 2000), as higher nitrogen content is thought to induce faster development and higher egg production in *Panonychus (Metatetranychus) ulmi* (Breukel and Post 1959; Crooker 1985; but see English-Loeb 1989). Lewontin (1965), and Wrensch and Young (1975), showed that small differences in the rate of development generally lead to large differences in fecundity. This argument may also explain the higher fecundity of *T. ludeni* on *A. cruentus*.

Although total female fecundity (measured as total number of eggs/female) was higher on *A. cruentus* compared with *S. macrocarpon*, life table parameters such as number of progeny per female (i.e. number of adult offspring per female), survival of immatures to the adult stage, proportion of females in the progeny and net reproductive rate were higher on *S. macrocarpon* than on *A. cruentus*. This suggests that egg viability was lower on *A. cruentus* than on *S. macrocarpon*. Indeed many eggs of *T. ludeni* on *A. cruentus* failed to hatch (Table 2), and juvenile mortality was also observed to be higher on *A. cuentus* than on *S. macrocarpon* (Table 3). The temporal fluctuations of female production with age of parents showed a clear decrease, especially on *A. cruentus*. This is likely due to the fact that as the females age, they run out of sperm and start producing more males, a common phenomenon with arrhenotokous species (Wrensch 1985; Roy et al. 2003). With *S. macrocarpon*, the decline was not pronounced, suggesting once more that *S. macrocarpon* is more suitable for *T. ludeni* than *A. cruentus*. The intrinsic rates of increase (*r_m_*) confirm these suggestions, as it was higher on *S. macrocarpon* than on *A. cruentus*. However, the data found in this study are lower than the 0.273 observed on cotton at 28°C by da Silva (2002), but fall within the value range generally observed for *Tetranychus* species (Sabelis 1985).

The high mortality recorded during the immature stages on *A. cruentus* may be due to its chemical composition as it may contain some so-called digestibility reducers known to exert sublethal effects by impairing herbivores' growth. Several studies have shown that population density and fecundity of various tetranychid mites on various host plants depend on plant quality (Kerguelen and Hoddle 2000). Some chemical elements such as nitrogen, phosphorus and potassium, present in varying amounts in the plant may be responsible for poor growth and high mortality in tetranychid populations (Price et al. 1980; Puttaswamy 1980; Puttaswamy 1981; Crooker 1985). Nitrogen fertilization and/or high nitrogen content of the plant has a positive effect on life-history parameters of spider mites. In contrast, there is a negative correlation between the rate of population increase (*r_m_*) of *T. urticae* on bean leaves and the potassium content of the leaves (see review by Wermelinger et al. 1991). The chemical composition of *A. cruentus* shows that its potassium content is much higher than that of *S. macrocarpon* (Bukenya and Bonsu 2004; Grubben 2004), and this might well be responsible for the lower *r_m_* of *T. ludeni* on *A. cruentus*. Furthermore, some secondary plant metabolites such as phenolic compounds are also known to adversely affect pathogens and insect/mite populations (Wermelinger et al. 1991), and this may also be another reason for the higher immature mortality of *T. ludeni* on *A. cruentus* compared with *S. macrocarpon*.

The population of *T. ludeni* used in these experiments came from *S. aethiopicum*, a solanaceous species close to *S. macrocarpon*. These mite populations might have, therefore, developed an adaptation to solanaceous species and this could enhance their performances on *S. macrocarpon* compared with *A. cruentus*, an Amaranthaceae. The higher net reproductive rate and the shorter doubling time of *T. ludeni* on *S. macrocarpon* may explain the relatively higher densities of *T. ludeni* generally observed on solanaceous plants in the field (Adango 2005). It is widely acknowledged that although spider mites are highly polyphagous, they accept and perform differentially on diverse host plant species (van den Boom et al. 2003; Greco et al. 2006).

This study shows that population density and fecundity of *T. ludeni* are dependent on host-plant quality. Demographic parameters of this mite pest are better on *S. macrocarpon* than on *A. cruentus*, suggesting that *S. macrocarpon* is more suitable to *T. ludeni* than *A. cruentus*. This differential suitability of host plants to the mite is an important factor to consider while exploring IPM solutions for *T. ludeni*. For example, intercropping and/or rotation of *A. cruentus* and *S. macrocarpon* could be used to break the dissemination of *T. ludeni* within a vegetable farm. Moreover, a full knowledge of the chemical composition of potential host plants and/or non-host plants of the mite pest, and of how this can affect the growth of the mite might be an important key for developing biopesticides for a cheaper and environmentally safer control of mite pest on vegetable farms in Africa.
